# Dissemination of Drinking Water Contamination Data to Consumers: A Systematic Review of Impact on Consumer Behaviors

**DOI:** 10.1371/journal.pone.0021098

**Published:** 2011-06-27

**Authors:** Patricia J. Lucas, Christie Cabral, John M. Colford

**Affiliations:** 1 School for Policy Studies, University of Bristol, Bristol, United Kingdom; 2 School for Social and Community Medicine, University of Bristol, Bristol, United Kingdom; 3 Division of Epidemiology, School of Public Health, University of California, Berkeley, California, United States of America; Ludwig Maximilian University of Munich, Germany

## Abstract

**Background:**

Drinking water contaminated by chemicals or pathogens is a major public health threat in the developing world. Responses to this threat often require water consumers (households or communities) to improve their own management or treatment of water. One approach hypothesized to increase such positive behaviors is increasing knowledge of the risks of unsafe water through the dissemination of water contamination data. This paper reviews the evidence for this approach in changing behavior and subsequent health outcomes.

**Methods/Principal Findings:**

A systematic review was conducted for studies where results of tests for contaminants in drinking water were disseminated to populations whose water supply posed a known health risk. Studies of any design were included where data were available from a contemporaneous comparison or control group. Using multiple sources >14,000 documents were located. Six studies met inclusion criteria (four of arsenic contamination and two of microbiological contamination). Meta-analysis was not possible in most cases due to heterogeneity of outcomes and study designs. Outcomes included water quality, change of water source, treatment of water, knowledge of contamination, and urinary arsenic. Source switching was most frequently reported: of 5 reporting studies 4 report significantly higher rates of switching (26–72%) among those who received a positive test result and a pooled risk difference was calculate for 2 studies (RD = 0.43 [CI0.4.0–0.46] 6–12 months post intervention) suggesting 43% more of those with unsafe wells switched source compared to those with safe wells. Strength of evidence is low since the comparison is between non-equivalent groups. Two studies concerning fecal contamination reported non-significant increases in point-of-use water treatment.

**Conclusion:**

Despite the publication of some large cohort studies and some encouraging results the evidence base to support dissemination of contamination data to improve water management is currently equivocal. Rigorous studies on this topic are needed, ideally using common outcome measures.

## Introduction

Access to safe drinking water is essential for health and, some argue, a basic human right [1]. Drinking water contaminated by human and animal feces contributes significantly to diarrheal diseases, a major cause of death in developing countries [2]. Children under five and immuno-compromised adults are particularly vulnerable [3]. A recent systematic review estimates diarrhea related annual mortality in children under five to be 1.9 million globally, of which 78% (1.5 million) occurred in the developing world [4]. Chemical contamination of drinking water presents risks to a smaller global population but is a serious human health hazard for those affected [5]. Arsenic and fluoride in drinking water present the greatest health risks [6], for example as many as 77 million people may be affected by Arsenic contamination of drinking water in Bangladesh [7].

Much can be done to reduce the burden of disease attributable to unsafe drinking water. Estimates from meta-analyses suggest water quality interventions can reduce rates of child diarrhea morbidity by 42%, while water supply interventions have little effect [8], [9]. There are two principal routes to improving the quality of consumed water: improving water quality at the source by better community management, or improving water quality in the home through ‘point-of-use’ (POU) treatment. The high cost of repeated multiple acts required to maintain high water quality combined with a low perceived threat have been observed as a possible explanation for the long- term failure of safe water interventions [10]. Moreover, in the developing world in locations where formal water supplies are of variable quality [11] and informal supplies abound, water consumers are unlikely to know which sources are contaminated and when POU treatment is necessary. The idea has emerged that testing water for contaminants (both chemical and microbiological) and disseminating the results to consumers might promote behavior change by increasing awareness of the threat and informing communities about the difference in water quality between different sources. The popularity of this approach is exemplified in UNICEF's ‘Water Alert’ game [12] which teaches young people that “testing the water, sharing the results and warning the villagers…is critically important” to protecting the health of the village.

This paper describes a systematic review of the literature examining the efficacy of the use of water quality information dissemination at changing either household or community water management behavior. The primary health outcomes of interest result from improvements in water quality, and are downstream to a number of interim outcomes which must occur:

1. Knowledge of water contamination (consumers must know the results of tests undertaken before responding to them)
2. Behaviors undertaken to improve water consumed
  - Switching to the safest (or least contaminated) sources
  - POU water treatment
  - Improved management of shared water sources, usually through source treatment
3. Water quality improvements since health improvement can only be expected if the actual quality of the water improves

The behavioral changes required will vary according to the contaminant identified and local context. For example, removal of arsenic from water is plausible [13] but rare whereas removal of pathogens by chlorination of either community or household water source is common and widespread.

The aim of the systematic review was to evaluate this literature to establish the evidence for impact of dissemination of water quality information about a) chemical contamination and b) microbial contamination on health outcomes, knowledge of risk, source switching, POU water treatment, source treatment, and water quality improvements.

## Methods

A protocol for this review was developed and reviewed by colleagues external to this team and is available on request from the first author. Reporting guidelines set out in the PRISMA statement are followed here [14].

### Searching

Seven bibliographic databases were searched during January 2010 (CENTRAL, MEDLINE, PsychInfo, EconLit, Compendex, LILACS, IndMed). Search strategies varied by database, but were structured to include terms for [drinking water] AND [water contamination] AND [test]. The full search strategy for Medline (Ovid online platform) is provided in Supporting Information File S1, and all further search strategies are available on request from the corresponding author. Particular efforts were made to locate unpublished reports using OpenSIGLE, relevant conference proceedings, Google searches and snowballing from known projects. Reference lists from included studies were screened for further studies and 5 non-systematic reviews were screened for further studies [15], [16], [17], [18], [19].

### Selection

The first 2,000 records were independently screened by 2 reviewers (CC & PL) for potential inclusion in the scoping review. Since agreement was high, 25% of the remainder were independently double screened. All studies identified as potentially relevant to the scoping review were then reviewed in full (independently by 2 reviewers) for inclusion in the systematic review. Any disagreements were settled through discussion and consensus.

Eligibility for inclusion was assessed against the following criteria:

- **P**opulations living in areas where chemical or microbiological contamination of drinking water posed a known health risk
- **I**nterventions in which drinking water contamination was tested and results disseminated to individuals or communities. Testing could take place at any local site (eg: private well or tap, shared well or tap, community source)
- **C**omparison. Only studies using alternate or no-treatment control groups were included
- **O**utcomes of interest were changes in: health, water source, water treatment, water quality, and knowledge of contaminant risk
- **S**tudy designs included were Randomized Controlled Trials (RCTs), Quasi-RCTs, Cohort Studies, Time series, and Controlled (including non-equivalent comparison groups) before and after studies

Exclusion criteria:

- Studies conducted in locations where water contaminants did not pose a significant public health threat (such as exposure to low concentrations of nitrites)
- Studies where general risks posed by unsafe water were highlighted, without dissemination of local contamination data following testing
- Studies where no outcomes of interest were collected or reported

### Validity assessment

Risk of bias was assessed using current guidance from the Cochrane Collaboration [20]. This tool considers bias in: sequence generation, allocation concealment, blinding, missing outcome data, selective outcome reporting, and “other sources of bias.” For non-randomized controlled studies included, we substituted comparability of groups at baseline and follow-up for security and concealment of randomization. In addition we assessed intervention integrity, ie the uniformity of the intervention delivery.

Studies were categorized as having a low, moderate, or high risk of bias using standard criteria for each study type as advised and disagreements resolved through consensus. The risk of bias will be reported separately for each study and for each outcome. All outcome data will be reported regardless of level of bias reported but where risk of bias is high this will be highlighted in our assessment of the strength of evidence.

### Data Abstraction

Abstraction was completed independently, in duplicate.

### Study characteristics

Abstracted study characteristics were population characteristics, drinking water supply, intervention details (type and frequency of water testing undertaken, methods of information dissemination, intervention duration and any co-interventions), nature of control or comparison group, and period of follow up.

Outcomes extracted were:

- health outcomes attributable to consumption of contamination water (e.g. diarrhea, flourosis, arsenicosis) assessed by occurrence of symptoms in study populations from self report or health care data and converted into risk difference (exposed – non-exposed) where possible
- water quality measured using any standard methods for assessing potability of drinking water usually through tests for presence/absence of microbial or chemical indicators or concentrations of contaminants. Reported here as risk difference for dichotomous, or Standardized Mean Difference for continuous, outcomes where possible
- source switching measured using self reported proportion of households changing their main water source. These data are categorical (e.g. switching to less safe, not switching, switching to safer) but may be restricted to dichotomous data (eg proportion of study population switching to a safer source). Risk difference for dichotomized data will be reported here where possible. Proportions within each switching category will also be reported where data are available
- water treatment (at source or point of use) measured using self reported water treatment, researcher observed water treatment or standard tests for water treatment (e.g. tests residual free chlorine. Reported here as risk difference for dichotomized data where possible (e.g. treated/not treated, sufficient/insufficient chlorine)
- correct knowledge of contamination risk among study communities before and after intervention. The proportion of study population correctly knowing the safety of sources was reported as a risk difference where possible

### Quantitative data synthesis

The study team agreed that the capacity for meta-analysis would depend on the heterogeneity of interventions, study types and outcomes available and was likely to be highly constrained. Therefore only subgroups and not meta-analytic strategy were planned in advance. These were: type of contamination, method of dissemination, level of contamination, and study design. We suggest that pooling of effect sizes will only be appropriate among studies of the same contaminant and using similar interventions. If data were to be available in later updates, continuous data would be pooled using inverse-variance methods. Methods for pooling risk differences would be determined by rate of events and study characteristics [20].

## Results

**Figure 1:**
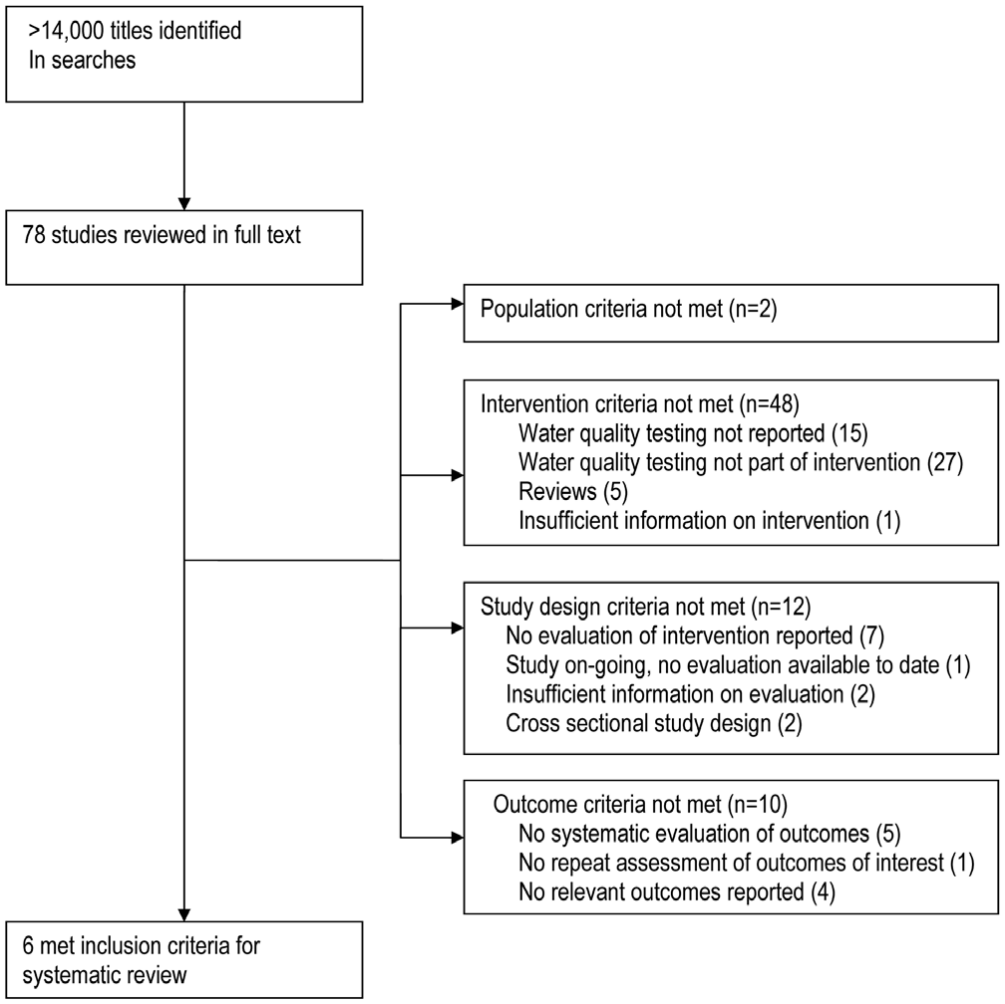
Flow chart of included/excluded studies.

More than 14,000 unique documents were located (including duplicates). Six studies (due to multiple publications, number of reports is larger than the number of studies) met the inclusion criteria for systematic review, see Figure 1 Flow chart of included/excluded studies. Excluded studies are shown in Supporting Information File S2.

### Excluded Studies

Twenty-two projects met intervention criteria, but not study design criteria. The variety of projects and studies identified confirms the interest in the use of water quality monitoring as part of community level public health activities. Since this is the first review of this body of literature, some readers may be interested in the range of these studies so further details are provided in Supporting Information Table S1.

### Study characteristics

The characteristics of included studies are summarized in Table 1. Four included studies concerned arsenic contamination [21], [22], [23], [24], [25], [26], [27], [28], [29], [30], [31], [32] and 2 concerned indicators for fecal contamination (*E. coli* or H_2_S-producing bacteria) [33], [34].

**Table 1:** Characteristics of Included Studies.

Publications/Reports	Population	Intervention	Comparison/control
*Arsenic Contamination Studies*			
Arsenic Policy Support Unit [21], [22]	BangladeshRural areas66% of wells >50 µg As/ltr^1^	Well testing and labeling as safe/unsafe	Comparison areas
HEALS Studies [23], [24], [25], [26], [27], [28]	BangladeshRural areas52% of wells >50 µg As/ltr72% of wells >10 µg As/ltr^2^	Well testing and labeling as safe/unsafe together with advice to switch wells and village level public education campaign	Comparison areas used in some analyses
Planning Alternatives for Change[30], [31]	BangladeshUrban area20% of sample water >100 µg As/ltr	Well testing and labeling as safe/unsafe/unknown together with advice to switch wells and public education campaign	Some comparison data, since not all those in the study areas received the intervention
Tarrozi [32]	Bangladesh62% >50 µg As/ltr	Well testing and household visits to inform householders of the actual level of contamination. Households were randomized to a message emphasizing a ‘gradient’ risk or a ‘binary’ risk message	Alternate treatment controls
*Microbiological Contamination Studies*			
Jalan and Somanathan [33]	New Delhi, India.Urban area60% of samples tested positive for fecal indicators	Tested household water for fecal indicators (H_2_S) & returned the test results (safe/unsafe) with advice and information on locally available purification methods	No treatment control
Luoto [34]	KenyaRural areas86.5% of household samples tested positive for fecal indicators	Source water and/or household stored water tested for fecal indicators (E Coli) and household informed of source and/or household water contamination results. Study also tested purification products, message ‘framing’ conditions, and ‘commitment’ messages.	Alternative treatment controls

1 Bangladesh Government safe limit for Arsenic in drinking water.

2 WHO safe limit for Arsenic in drinking water.

All the participants in the study groups were shown to be at high risk of consuming contaminated water. Among the studies of arsenic contamination the proportion of wells with arsenic levels higher than the Bangladeshi safety limit of 50 micrograms per liter (50 µ/L) varied between 20% [30] and 66% [21]. The WHO standard is lower (10 µ/L), and using this standard 44% of wells were unsafe [27]. In the two studies of microbiological contamination the WHO approved safety standard is zero presence of fecal indicators and 60% [33] and 86.5% [34] of water sources were judged unsafe against this standard.

Four were based in Bangladesh (all concerning arsenic contamination), one study in India and one in Kenya (both microbiological contamination). All studies used external groups (the research team or NGOs) to test the water and disseminate results and all required behavior change at the individual rather than community level.

The risk of bias in these studies was judged as moderate to high in most cases considering study design, sampling and missing information (see Table 2). Two recent studies were (at the time of searching) still only available as working papers and further data or analyses may be available in later publications [32], [34]. Only 3 studies used random allocation [32], [33], [34], and only one of these included a no-information control group [33]. All except 1 study (where delivery differed between areas [21], [22]) studies had good intervention fidelity.

**Table 2:** Included Studies Assessment of bias.

Reporting Study	Study Description & Sample Size	Assessment of bias
Arsenic Policy Support Unit [21], [22]	*Cohort of n = 4.5 million, from whom study sample recruited (approx n = 4453)*	High risk of bias, taking into account:1. Different samples were recruited at baseline and follow up. The post-intervention sample were wealthier and better educated.2. V Low response rates (1%) to some questions3. Response rates varied between areas
HEALS Studies [25]	Prospective Cohortn = 11746 (6512 using high arsenic wells)	Risk of bias was low/modest taking into account:1. Inclusion criteria restricts generalisability (married & lived in area >5 years2. Low attrition rates (n = 11280, 96%)3. Significant differences at baseline between groups partly accounted for in analyses
HEALS Studies [28]	Subsample of larger study [25] compared to neighboring areasIntervention n = 2680 (1089 couple interviews, 502 individual)Comparison n = 997 (500 couples)	High risk of bias taking into account:1. As for Chen et al 2007 [25]2. Significant differences at baseline between groups not accounted for in analyses.3. Intervention integrity and fidelity was good
Planning Alternatives for Change [30], [31]	Cohort of n = 300,000 , from whom n = 694 recruited	High risk of bias, taking into account:1. Participants self-identified as having been exposed to intervention or not2. Overall attrition high (n = 228, 44%) and differential attrition rates in those exposed to (64%) or not exposed to (27%) intervention3. Little data provided concerning possibility of bias in selection or confounding factors4. Intervention integrity was high
Tarozzi [32]	Randomized trial of different forms of intervention delivery^1^, all arms received water quality information.668 households within 45 villages.	Moderate risk of bias, taking into account:1. Randomization process secure, but unit of randomization & some unit of outcome differed2. No control group for information element3. No significant differences between clusters4. Attrition low (follow up n = 605, 91%)5. Exclusion criteria (eg recent change of well) restricts generalisability somewhat
Jalan and Somanathan [33]	Randomized controlled trial (no treatment control)n = 1006 households	Moderate risk of bias, taking into consideration:1. Method of randomization not described2. Group allocation secure on variables checked by authors, although important variables not assessed (e.g. wealth, education)3. Low attrition rates, differential attrition accounted for in analysis
Luoto [34]	Randomized trial of multiple intervention conditions^2^.400 Households (ave household size 6) in 28 villages	Risk of bias was high taking into account1. Study was not blinded2. No control group for information element3. Wealthier households were more likely to be assigned to early information sharing4. Attrition low (360 HHs, 92.5%) & equal across 3 information arms (96%, 97% & 95%)

1 Emphasizing binary or continuous assessment of safety.

2 3 information conditions: Informed about common source and household stored water quality at times 1 and 2 (not at time 0), Informed about common source at times 1 and 2 and household stored water quality at time 2 (not at time 0) or informed about common source at time 2 (not at time 1 or 0) (i.e. a waiting list control group only). Villages were randomly assigned to information treatment, with 133, 123 and 130 households in each arm.

There was only one occasion where sufficient data were available on common outcomes using compatible study designs to allow meta-analysis. A narrative (or qualitative) account of findings is provided here. Where possible (i.e. when data allow) outcome data has been converted into effect sizes, but are mostly reported as presented in the original studies. Unpublished data were available for two studies, study authors for an RCT of microbial contamination in India have made their data publicly available [35], the first author of a second study provided additional information on source switching behavior [36]. Findings are summarized in Tables 3 and 4.

**Table 3:** Impact on Switching to Safer Water Sources.

Reporting Study	Findings
HEALS [25] 1	2 year follow up.58.1% of those with wells labeled unsafe switched to a different well, compared to 17.3% of those with wells labeled safe.When this is broken down by level of contamination, Rate Ratio of switching to a known safe is significantly higher among those with higher Arsenic concentration after adjusting for baseline characteristics including age and sex (e.g. 100–299 As µg/ltr RR = 1.38, 95% CI 1.23–1.55, n = 3433) and among those with unsafe wells who had received the education campaign (RR 1.84, 95% CI 1.60–2.11, n = 4894).Within intervention areas Risk Difference for switching comparing those with a safe well to those with an unsafe well is 0.08 [CI 0.06,0.09].
HEALS [28]	6–12 months follow up.In arsenic mitigation program areas, 60% of those with an unsafe well had switched to different well and 14% of those with a safe well. In comparison areas 8% of people had switched to different well.Within intervention areas Risk Difference for switching comparing those with a safe well to those with an unsafe well is 0.46 [CI 0.43,0.49].Risk difference is estimated at 0.32 [CI 0.30,0.32] comparing intervention and comparison areas however since this sample includes some couples observations are not independent.
Tarozzi [32]	9 months follow up.Within an intervention area 34% of those with an unsafe well switched well compared to 8% of those with a safe well (significance not tested). Emphasis on higher levels of arsenic in a ‘gradient’ message did not increase likelihood of change above the binary message.Risk difference for switching comparing those with a safe well to those with an unsafe well is 0.28 [0.22,0.34]^2^.
APSU [21], [22]	Approx 6 months follow up.Both studies report participants switching away from unsafe wells.However, data are not reported here because low reporting rates means we can't ascertain reliability (eg baseline n = 2,357, only 209 responded to testing question of whom only 44 report both a positive results and answered question about switching.
PAC [30], [31]	The numbers using water from wells labeled safe at follow up (n = 302) reported as significantly higher (p<0.01 no test statistics provided) among those self reporting as having received the intervention than those not; for drinking 56.6% and 85.1%, for cooking 53.9% and 74.3% and for soaking cereal for breakfast 45.6% and 70.3% respectively.Risk difference could not be calculated from available data.
Luoto [34]	6 month follow up.The proportion of households whose untreated stored water show no sign of fecal contamination (E. coli <10/100 mL^3^) increased by 0.07% (SE 0.04, p<0.1). The author concludes this implies switching to sources that are less contaminated.Risk difference could not be calculated since no comparison data are available.

1 Early findings reported in Opar et al 2007 not reported here).

2 Additional data provided by study author.

3 Most Probably Number of Colony Forming Units.

**Table 4:** Impact on Other Outcomes.

Outcome	Reporting Studies
**Urinary Arsenic**	2 year follow up data HEALS [25].Decrease in creatinine-adjusted Urinary Arsenic of −109 µg/L (sd = 319.9, n = 5812) among those informed with wells labeled unsafe compared to −6.2 µg/L (sd = 107.4, n = 4814) among those with a safe well, p<0.01.SMD = −0.42 [CI −0.45,−0.35].
**Water quality**	6 month follow up data Luoto [34].Household water quality improved significantly (−0.6 log E Coli, SE = 0.17, 1357 observations, p<0.01) among those informed of source contamination.Household water quality did not improve significantly (−0.11 log E Coli, SE = 0.18, p>0.1) among those informed of household contamination.Means change and SD not available for comparisons therefore SMD not calculable here.
**Water treatment**	3 month follow up Jalan [33] 1.The intervention group purified less at baseline (intervention 45.7% n = 527, control 48.9% n = 479), but equally (47%) at follow up (control n = 477, intervention n = 520).Risk difference at baseline for treat/not treat = −0.03 [−0.09,0.03].Risk difference at follow up for treat/not treat = 0 [−0.06,0.06].8.2% of those informed that their water was contaminated began purification compared to 3.2% of those informed their water was safe and 3.8% of controls.6.1% of those informed that their water was contaminated stopped purification compared to 3.2% of those informed their water was safe and 2.1% of controls.
	6 month follow up data Luoto [34].Authors state that “sharing of common source water quality information was associated with a 8–13 percentage point rise in rates of POU adoption…Sharing of personalized water quality information does not further increase POU usage.” Contributing data are not provided therefore risk difference cannot be calculated, although effect sizes plotted on a graph show increases.
**Knowledge of contamination**	In APSU study knowledge of the meaning of well labels was reported as higher after the intervention (25% more correctly answered the question t0 n = 858, t1 n = 1082) [21], [22].Risk difference not calculable as only change score reported.
	In PAC study 80% of the ‘program influenced’ (ie. among those who recalled intervention) vs 25% of Non-program influenced people knew meaning of red labeled wells [30], [31].Risk difference not calculable as sample size for this survey question were not reported.
	6–12 month follow up HEALS [28].98% of 2071 respondents in intervention areas said they knew their well safety, compared to 20% of those in a comparison area although we can't verify the accuracy of this knowledge.Risk difference not calculable as sample size of responders in control areas not reported.
	In Tarozzi's study 79% (SE = 0.02, n = 587, p<0.01) of respondents knew the water safety, 3% knew actual arsenic level. Those who have an unsafe well less likely to know the safety (26% SE = 0.03, n = 587, p<0.01). [32]Risk difference not calculable as no comparison data are available.

1 Data reported here is a reanalysis of raw data provided by study authors who report different study outcomes.

### Impact on Source Switching

The strongest evidence was for source switching in response to arsenic contamination information, with 4 studies reporting higher rates of switching (26–52%) in households previously drinking from contaminated wells (3 at statistically significant levels) [25], [30], [32], [37]. Three studies compared within a cohort between those using safe or unsafe wells at baseline [21], [25], [32] and one between participants and non-participants in an education campaign [30]. In addition, comparison data from an area neighboring the Health Effects of Arsenic Longitudinal Study (HEALS) (group where well labeling had not yet taken place showed lower switching rates (8%) than among those with safe (14%) or unsafe (60%) wells in intervention areas [28]. In Kenya, a modest increase in the proportion of pre-treated stored water with low levels of microbial contamination (<10 colony forming units (Cfu) 100/ml) is taken to infer source switching [38].

Although 5 studies report rates of switching a common estimate of effect (Risk Difference) could only be calculated for two studies both of which considered arsenic contamination of drinking water in Bangladesh, both with low/moderate risk of bias. Tarozzi and colleagues [32] showed that those with unsafe wells were more likely to switch sources 9 months after receiving the information [RD 0.28, CI 0.22–0.24]. In the HEALS studies 6–12 months after intervention those in the study areas were more likely to switch source than those in the comparison areas [RD 0.32, CI0.30–0.32] and within comparison areas those with unsafe wells were more likely to switch source than those with safe wells [RD 0.46 CI 0.43–0.49] [28]. At two year follow up differences between those with safe and unsafe wells were modest but remained [RD 0.08 CI 0.06–0.09] [25]. The data comparing switching rates between those with safe and unsafe wells at 6–12 month follow-up can be pooled to show a significant difference in rates of switching [RD 0.43 CI0.4–0.46] suggesting that 43% more of those who were informed that their well was unsafe switched sources relative to those who were told their water source was safe. The strength of evidence provided by these findings is low because the comparison made is between non-equivalent groups; those who have an unsafe well may be dissimilar to those who have a safe well introducing a potential source of bias.

### Health Effects

Only one study reported health outcomes from information sharing. The HEALS study reports creatinine adjusted urinary arsenic in a cohort whose wells had been labeled to identify safe/unsafe levels of arsenic. They report a significant reduction of urinary arsenic among those using unsafe wells at baseline (109 vs 6.2 µ/L, effect size 0.86, 95% CI 0.18,1.5 unadjusted for baseline differences between groups, risk of bias in study judged to be low/moderate). This represents a Standardized Mean Difference of −0.42 [CI −0.45,−0.35] between groups, favoring those who had been informed that their wells were unsafe.

### Water Quality

Only one study reported water quality as an outcome. Luoto compared the levels of *E.coli* in household water (colony forming units per liter of water: cfu/100 ml), comparing pre and post intervention only [34]. This study used alternate interventions presented sequentially in random order (i.e. all groups received source and household water quality information at some time) although this study was judged to have a high risk of bias considering differences at baseline between groups. A significant reduction in *E.coli* in household water following dissemination of source water quality results was reported (mean reduction of 0.6 log cfu/100 ml SE = 0.17, n = 1357, p<0.01), but not following information about household water quality (mean difference 0.11 log cfu/100 ml, SE = 0.18, p>0.1).

### POU Water treatment

POU treatment is the primary outcome for both the included studies of microbiological contamination, both of which employed random allocation. In Kenya, Luoto states that POU rose significantly after being informed about source water quality, but not after being informed about household water quality [34]. In India in a study judged to have a moderate risk of bias, there was a reduction in purifying frequency of 1.5% among the control group, and an increase of 1.8% among intervention, however 48.8% of both groups never purified (original data made available to review authors and reanalyzed here) [33]. This new analysis showed a significant 2 way (χ^2^ (df 13) = 36.07, p.001) but not 3 way (χ^2^ (df 4) = 6.52, p = 0.16) interaction. Interactions were significant for both Group × outcome (χ^2^ (df 4) = 16.34, p = 0.03) and Test result × outcome (χ^2^ (df 4) = 11.21 , p = 0.02), but not for group × test result (χ^2^ 0.76 (df 1), p = 0.23). The groups were unbalanced; members of the experimental group were significantly more likely to receive a positive test result, significantly more likely to start purifying but also to stop purifying (10.3%) than the control group. It is difficult to say with certainty what the effect of the provision of contaminant information was in this case.

### Knowledge of the contamination level

Knowledge of the contamination level of their water source was collected as an interim outcome in some studies. Where this outcome is reported, increases in knowledge of between 25–78% following intervention were observed [21], [28], [30] although all studies were judged to have a high risk of bias.

### Planned Subgroups

The only common outcome reported between studies of chemical and microbiological contamination was source switching, where evidence of source switching was provided by four studies of arsenic contamination [21], [22], [25], [28], [30], [31], [32], [34] and one study of microbiological contamination [34]. Given limited data availability it was not possible to compare impact according to contaminant risks discussed.

Similarly, it is difficult to draw conclusions from these studies regarding method of information delivery or level of contamination. None of the studies compared between modes of communication or personnel delivering results. Most studies used a combination of approaches including house-to-house visits, public education campaigns, and public displays of information (such as well labeling) but did not compare between strategies. The two most recent studies are an exception to this, where researchers compared different strategies for information dissemination. In Bangladesh binary and ‘degrees of risk’ information about arsenic contamination were compared [32], and in Kenya message framing and sharing of source vs household water quality were compared [34]. One other study shared information on levels of contamination alongside a binary safe/unsafe message but did not compare approaches [25]. Positive framing of messages (i.e. emphasizing health benefits rather than health risks) increased the likelihood of POU [34] . Information about level of risk had the largest impact on behavior at the boundary of safe/unsafe level: those who were just above the risk level were more likely to switch source if they received the gradient message than those receiving binary information, but at higher levels those receiving binary information were more likely to switch [32].

The three studies employing randomization were also the three most recent studies. Unfortunately the data collected regarding *our* outcomes of interest were limited. All three studies conclude that the provision of water quality information was successful in promoting behavior change, although Luoto notes this only held true for information about source, not household, water in her study [34].

## Discussion

The search strategy for the review was deliberately wide to gather studies from across disciplines (health, engineering, economics and psychology) for any drinking water contaminant risks. We sought out published and unpublished sources. To our knowledge this review provides the most comprehensive collation of studies of this type yet published, and demonstrates the widespread interest in this intervention. Our review has identified the strengths and limitations of the existing evidence and indicated how future studies might report relevant exposures and outcomes in a way to allow for proper meta-analysis.

### Limitations

The literature in this field is not well established and no studies with low risk of bias and complete reporting of outcome data of interest to this review were found. One limitation of this review is its reliance on narrative synthesis in response to heterogeneity in study designs and outcomes included. This is a common difficulty in public health research and particularly so in developing country public health [39], [40], and we believe that narrative synthesis is an appropriate strategy in this context.

Some bodies of literature are likely to have been missed by our approach to searching. For example, studies conducted within water engineering documenting incremental changes in water management systems may not have been retrieved. Since our aim was to explore the effects of information dissemination to communities or consumers we do not believe such studies would have met our inclusion criteria. Similarly, the rich literature on risk communication [41], [42], [43] which considers how information is most effectively presented is not included. This literature should be drawn on to design and interpret interventions in the field.

### Policy Context

Considering all studies meeting the intervention criteria (including those excluded because of study design) this review demonstrates that the use of water testing and dissemination as a tool for behavior change, particularly with respect to microbiological contamination, is being promoted ahead of the evidence of impact. This adoption is often large scale; in just one state in India (Andhra Pradesh) 24,000 field kits (chemical parameters) and 13,50,000 H_2_S (Hydrogen Sulfide) tests have been distributed to Panchayats (village level government) in an effort to introduce community level monitoring of their water supply [44]. The evidence is encouraging, but not yet conclusive that this is an effective means of changing behavior. Twelve of the excluded projects aimed to promote better community management of their water supply [29], [45], [46], [47], [48], [49], [50], [51], [52], [53], [54], [55], [56], [57] and improvements in local management are widely reported in these studies [29], [50], [53], [54], [58], [59], although these assertions are not always accompanied by supporting data.

### Behavior Change

The studies reported here all attempt to change behavior by using information about contaminants in water as a lever. This solution certainly has some face validity, making visible hidden health risks. However, in order to fully understand the likely impact of this intervention we should consider what information, disseminated how, and in which contexts are most likely to lead to behavior change. Only one study [34] used an explicit theoretical model to design the format of the intervention. Evidence suggests that such theoretically driven interventions will have higher success rates [60] and best practice in methods for evaluating complex interventions in health suggest the importance of a broad approach to evaluation informed by both theory and context [61], [62].

Many studies in the water and sanitation field highlight the importance of social and cultural factors [63] and the complexity of behavior change required to improve community [64] or household water supplies. Gender, poverty, stigma, convenience and local social structures were identified as key social factors determining the likelihood of change in the arsenic mitigation programs in Bangladesh [25], [31]. Programs were also thought more likely to succeed where there was a local history of self-mobilization and/or strong local leadership on the issue [31].

This review has highlighted many gaps in the evidence to date. We have identified 4 key issues to consider in future studies:

1. The need for evidence of impact using robust methods (e.g. random allocation of study participants, use of non-intervention control groups)
2. The format of information provided (eg source and/or household, binary or continuous, risk or safety messages)
3. The methods of information dissemination
4. The use of community level interventions and outcomes

The need for such studies is greater in the dissemination of microbiological, rather than chemical contamination both because the scale of the health threat is larger and because of the smaller number of studies in this area. In the absence of randomized impact evaluations, ongoing projects could provide data on elements of implementation, behavior change and context. Any future evaluation should be informed by a careful consideration of the specific causal pathways implied by behavioral models [65], [66], [67] to ensure that moderating and mediating outcomes are assessed.

### Conclusions

This systematic review confirms a growing interest in the use of dissemination of water contamination information to promote behavior change, particularly with respect to the provision of H_2_S to communities for self-testing of fecal contamination. Large cohort studies of arsenic mitigation programs in Bangladesh suggest that consumers were more likely to change wells if they were informed which were contaminated with arsenic but the evidence base is currently equivocal since there is not robust comparison data from the groups not receiving information. Our ability to draw strong conclusions is limited by the nature of the evidence collected to date where few studies have used robust control or comparison groups; rigorous studies on this topic are needed in which common designs and outcome measures are used.

## Supporting Information

File S1 Full search strategy in Medline.
(DOC)

File S2 Excluded Studies.
(DOC)

Table S1 Detail of Projects Meeting Intervention Criteria Not Included in Systematic Review.
(DOC)
