# Supplementary material for: Dissemination of Drinking Water Contamination Data to Consumers: A Systematic Review of Impact on Consumer Behaviors
Source: PLoS One. 2011 Jun 27;6(6):e21098. doi: 10.1371/journal.pone.0021098 (PMC3124476; doi:10.1371/journal.pone.0021098)
Supplement: File S1 — Full search strategy in Medline. (DOC) [file pone.0021098.s001.doc]

### S1 Full search strategy in Medline

1 Water Pollutants, Chemical/ or Water Pollutants/ or Water Supply/ or drinking water.mp. or Water Microbiology/ or Water Pollution, Chemical/

2 potable.mp.

3 test$.mp.

4 1 or 2

5 3 and 4

6 controlled clinical trial/ or randomized controlled trial/ or comparative study/ or evaluation studies/

7 random allocation.mp. or Random Allocation/

8 (clin$ adj25 trial$).ti,ab.

9 ((singl$ or doubl$ or trebl$ or tripl$) adj25 (blind$ or mask$)).ti,ab.

10 random$.ti,ab.

11 Research Design/

12 quasi-random$.mp.

13 cohort studies/ or longitudinal studies/ or follow-up studies/ or prospective studies/

14 Single-Blind Method/

15 Double-Blind Method/

16 clinical trial.pt.

17 Comparative Study/

18 Evaluation studies.mp. or Evaluation Studies/

19 interrupted time series.mp.

20 intervention$.mp. or Intervention Studies/

21 evaluation stud$.mp.

22 evaluat$.mp.

23 6 or 7 or 8 or 9 or 10 or 11 or 12 or 13 or 14 or 15 or 16 or 17 or 18 or 19 or 20 or 21 or 22

24 5 and 23
