# Supplementary material for: Dissemination of Drinking Water Contamination Data to Consumers: A Systematic Review of Impact on Consumer Behaviors
Source: PLoS One. 2011 Jun 27;6(6):e21098. doi: 10.1371/journal.pone.0021098 (PMC3124476; doi:10.1371/journal.pone.0021098)
Supplement: File S2 — Excluded Studies. (DOC) [file pone.0021098.s002.doc]

## S2 Excluded Studies

Reason for exclusion

## *Population criteria not met (2)*

## Schubert 1999 [1]

Poe & Bishop 2001

*Water quality testing not reported (15)*

Ali 2003 [2]

Alsop 2005 [3]

Bukenya 2008

deWilde 2008 [4]

Doe 2004 [5]

Gouveia 2007 [6]

Gungoren 2007 [7]

Hight 2006 [8]

Hite 2002 [9]

Lynch 1994 [10]

Madanat 1993 [11]

McConnell 2000 [12]

Syme 1993 [13]

Vieira 2005 [14]

Fonseca 2002 [15]

*Water quality testing not part of intervention (27)*

Abbas 2004 [16]

Alberini 1996 [17]

Amacher 1997 [18]

Bitto 1992 [19]

Colindres 2008 [20]

Colindres 2007 [21]

Egorov 2003 [22]

GRI undated [23]

Gupta 2007 [24]

Heinonen-Tanski 2007 [25] / COSI 2006 [26]

Isley 1978 [27]

Jabu (undated) [28]

Jakariya 2007 [29]

Janmaat 2007 [30]

Joshi 2003 [31]

Kaspar 1992 [32]

Khan 2004 [33]

Khan 2007 [34]

Kremner 2006 [35]

Kremner 2007 [35]

Martin undated [36]

May 2006 [37]

Nala 2003 [38]

Quick 2003 [39]

Susheela 2002 [40]

Susheela 2007 [41]

Tiwari 2004 [42]

*Reviews (5)*

Russel 2001 [43]

Ukoli-Onodipe 2003 [44]

Water Aid ANAISU 2002 [45]

WHO 2003 [46]

Zwane 2007 [47]

*Insufficient information on intervention (1)*

Nanan 2003 [48]

*No evaluation of intervention reported (7)*

Klink 2007 [49]

Shingles 2008 [50]/ Malteser International [51]

Nair 2008 [52]

WHO - OCHA 2008 [53]

Govt of India - Orissa Govt 2007 [54] / Uttarancha Govt 2005 [55]

UNICEF Malawi 2008 [56]

UNICEF Tajikistan 2008 [57]

*Study on-going, no evaluation available to date (1)*

POUZN project [58]

*Insufficient information on evaluation (2)*

NIOEH 2007 [59]

UNICEF Uzbekistan 2003

*Cross sectional study design (2)*

Hadi 2003

Schoenfeld 2005

*No systematic evaluation of outcomes (5)*

Colilert trials in Aboriginal communities (Byleveld 2000 [60]; Clarke et al 2002 [61]; Barnett et al 2004 [62]; Cooke & Sharp 2007 [63])

GRI undated [23]

IDRC 1996 [64]

Live & Learn 2006 [65]/ van der Tak [66]

Sanchez & Dutka 1998

*No repeat assessment of outcomes of interest (1)*

Howard 2002

*No relevant outcomes reported (4)*

Tet Nay Tun 2003 [67]

Venkatachalam, 2008 [68]

Shrestha 2003 [69]

Mimi [70]

References for all excluded studies

1. Schubert C, Knobeloch L, Kanarek MS, Anderson HA (1999) Public response to elevated nitrate in drinking water wells in Wisconsin. Archives of Environmental Health 54: 242-247.

2. Ali SM, ul-Haq R (2003) The Relationship between the WES Interventions and the Incidence of Diarrhoea. Pakistan Development Review 42: 555-567.

3. Alsop R, Kurey B Local Organizations in Decentralized Development: Their Functions and Performance in India.

4. deWilde CK, Milman A, Flores Y, Salmeron J, Ray I (2008) An integrated method for evaluating community-based safe water programmes and an application in rural Mexico. Health Policy and Planning 23: 452-464.

5. Doe SR, Khan MS (2004) The Boundaries and Limits of Community Management: Lessons from the Water Sector in Ghana. Community Development Journal 39: 360-371.

6. Gouveia C, Nicolau R, Ferreira F, Camara A (2007) Collaborative monitoring of chlorine flavours in drinking water. Water Science & Technology 55: 77-84.

7. Gungoren B, Latipov R, Regallet G, Musabaev E (2007) Effect of hygiene promotion on the risk of reinfection rate of intestinal parasites in children in rural Uzbekistan. Transactions of the Royal Society of Tropical Medicine & Hygiene 101: 564-569.

8. Hight J, Ferrier G Building Capacity to Monitor Water Quality: A First Step to Cleaner Water in Developing Countries.

9. Hite D, Hudson D, Intarapapong W (2002) Willingness to Pay for Water Quality Improvements: The Case of Precision Application Technology. Journal of Agricultural and Resource Economics 27: 433-449.

10. Lynch M, West SK, Munoz B, Kayongoya A, Taylor HR, et al. (1994) Testing a participatory strategy to change hygiene behaviour: face washing in central Tanzania. Transactions of the Royal Society of Tropical Medicine & Hygiene 88: 513-517.

11. Madanat S, Humplick F (1993) A Model of Household Choice of Water Supply Systems in Developing Countries. Water Resources Research 29: 1353-1358.

12. McConnell KE, Rosado MA (2000) Valuing Discrete Improvements in Drinking Water Quality through Revealed Preferences. Water Resources Research 36: 1575-1582.

13. Syme GJ, Williams KD (1993) The Psychology of Drinking Water Quality: An Exploratory Study. Water Resources Research 29: 4003-4010.

14. Vieira JMP (2007) Water safety plans: Methodologies for risk assessment and risk management in drinking water systems. IAHS-AISH Publication: 57-67.

15. Fonseca C, Bolt E (2003) How to support community management of water supplies: Guidelines for managers: Koninklijk Instituut Voor de Tropen

16. Abbas M, Schlosser DIW (2004) Water quality monitoring of improved water delivery systems in Northern Pakistan. 30th WEDC International Conference: People-centred approaches to water and envrionmental sanitation. Vientiane, Lao PDR: WEDC.

17. Alberini A, Eskeland GS, Krupnick A, McGranahan G Determinants of Diarrheal Disease in Jakarta.

18. Amacher GS, Feather PM (1997) Testing Producer Perceptions of Jointly Beneficial Best Management Practices for Improved Water Quality. Applied Economics 29: 153-159.

19. Bitto AO, Kale OO, Oduntan SO (1992) Epidemiological survey of an outbreak of gastroenteritis in a rural community in Oyo State. West African Journal of Medicine 11: 34-38.

20. Colindres R, Mermin J, Ezati E, Kambabazi S, Buyungo P, et al. (2008) Utilization of a basic care and prevention package by HIV-infected persons in Uganda: AIDS Care Vol 20(2) Feb 2008, 139-145.

21. Colindres RE, Jain S, Bowen A, Mintz E, Domond P (2007) After the flood: an evaluation of in-home drinking water treatment with combined flocculent-disinfectant following Tropical Storm Jeanne -- Gonaives, Haiti, 2004. Journal of Water & Health 5: 367-374.

22. Egorov A, Naumova E, Tereschenko A, Kislitsin V, Ford T (2003) Daily variations in effluent water turbidity and diarrhoeal illness in a Russian city. International Journal of Environmental Health Research 13: 81-94.

23. Global Resources Institute (2006) Sun Water: Water survey, water testing, and testing of solar disinfection in Lumbini, Nepal.: Global Resources Institute.

24. Gupta SK, Sheikh MA, Islam MS, Rahman KS, Jahan N, et al. (2008) Usefulness of the hydrogen sulfide test for assessment of water quality in Bangladesh. Journal of Applied Microbiology 104: 388-395.

25. Heinonen-Tanski H, Snel M, van Wijk-Sijbesma C, Quazi AR, Mathew K, et al. (2007) Assessing of environmental quality in six areas in India, Sri Lanka and Bangladesh. Environment, Development and Sustainability 9: 355-368.

26. COSI (2006) Innovative approaches to improve hygiene behavior and access to water in peri-urban coastal areas. Katugastota: COSI Foundation for Technical Cooperation.

27. Isely RB (1978) Community organisation approach to clean water and waste disposal in Cameroonian villages. Progress in Water Technology 11: 109-116.

28. Jabu GC Assessment and comparison of microbial quality of drinking water in Chikwawa, Malawi. Glasgow: University of Strathclyde. pp. 7.

29. Jakariya M, Vahter M, Rahman M, Wahed MA, Hore SK, et al. (2007) Screening of arsenic in tubewell water with field test kits: evaluation of the method from public health perspective. Science of the Total Environment 379: 167-175.

30. Janmaat J (2007) A Little Knowledge . . . : Household Water Quality Investment in the Annapolis Valley. Canadian Journal of Agricultural Economics 55: 233-253.

31. Joshi DD, Maharjan M (2003) Urban ecosystems and health in Kathmandu: community-based biological assessment of drinking water sources. Third International Conference on Environment and Health. Chennai, India: Department of Geography, University of Madras and Faculty of Environmental Studies, York University.

32. Kaspar P, Guillen I, Rivelli D, Meza T, Velazquez G, et al. (1992) Evaluation of a simple screening test for the quality of drinking water systems. Tropical Medicine & Parasitology 43: 124-127.

33. Khan AA, Paterson R, Khan H (2004) Modification and application of the Canadian Council of Ministers of the Environment Water Quality Index (CCME WQI) for the communication of drinking water quality data in Newfoundland and Labrador. Water Quality Research Journal of Canada 39: 285-293.

34. Khan MMH, Aklimunnessa K, Kabir M, Mori M (2007) Determinants of drinking arsenic-contaminated tubewell water in Bangladesh. Health Policy and Planning: 1-9.

35. Kremer M, Leino J, Miguel E, Zwane AP (2007) Spring Cleaning: Rural Water Impacts, Valuation, and Institutions. Harvard University & University of California, Berkeley. pp. 59.

36. Martin D, Knotsch C, Levessque B, Furgal C, Allen E, et al. Drinking water quality and climate change in Labrador: a pilot project for two Inuit communities. Quebec: Centre de Recherch du CHUL.

37. May D, LaFrenierre J (2006) Project Implementation Report Holistic Community Development in the Mae Yang Min Valley Year 2 - Water Delivery System and School Addition Mae Yang Min, Thailand. Durango: Engineers Without Borders, Fort Lewis College.

38. Nala NP, Jagals P, Joubert G (2003) The effect of a water-hygiene educational programme on the microbiological quality of container-stored water in households. Water SA 29: 171-176.

39. Quick R (2003) Changing community behaviour: experience from three African countries. International Journal of Environmental Health Research 13 Suppl 1: S115-121.

40. Susheela AK (2002) Fluorsis in developing countries: remedial measures and approaches. Proceedings of the Indian Natural Sciences Academy B68: 389-400.

41. Susheela AK (2007) Fluoride ingestion and health hazards with focus on anaemia in pregnancy and low birth weight babies: guidlines for rectification. 27th Conference of the ISFR. Bejing, China: Fluoride.

42. Tiwari DN (2004) Willingness to Pay for Improved Water Quality in Kathmandu. In: David Pearce CP, and Charles Palmer editor. Valuing the Environment in Developing Countries: Case Studies by (Paperback - 26 May 2004).

43. Russell CS, et al. (2001) Investing in water quality: Measuring benefits, costs and risks: IDB.

44. Ukoli G (2003) Designing Optimal Water Supply Systems for Developing Countries.

45. WaterAid (2002) Arsenic 2002: An overview of arsenic issues and mitigation initiatives in Bangladesh. NGO's Arsenic Information & Support Unit NAISU & Water Aid. 130 p.

46. WHO & OECD (2003) Assessing Microbial Safety of Drinking Water - Improving Approaches and Methods. Geneva: WHO & OECD,.

47. Zwane AP, Kremer M (2007) What Works in Fighting Diarrheal Diseases in Developing Countries? A Critical Review. World Bank Research Observer 22: 1-24.

48. Nanan D, White F, Azam S, Afsar H, Hozhabri S (2003) Evaluation of a water, sanitation, and hygiene education intervention on diarrhoea in northern Pakistan. Bulletin of the World Health Organization 81: 160-165.

49. Klink J (2007) E. coli contaminated drinking water in rural Uganda: Using results to make an impact. [Undergraduate]. Madison: University of Wisconsin-Madison.

50. Shingles K, Saltori R (2008) Community use of H2S (hydrogen sulphide) as a verification tool for water safety plans. 33rd WEDC International Conference Access to sanitation and safe water: global partnerships and local actions. Accra, Ghana: WEDC.

51. Malteser International (2008) From safe water and sanitation to good health. Cologne: Malteser International. 23 p.

52. Nair J (2008) H2S Water Testing Kit: A simple, easy to use water testing kit, developed for aboriginal communities.: ETC Murdoch University.

53. OCHA (2008) Darfur - OCHA-23: 10-Jul-08 UN Office for the Coordination of Humanitarian Affairs.

54. Orissa RDDGo (2009) Rural Water Supply: Highlights. Government of Orissa.

55. Govt. Uttaranchal (2005) Uttaranchal rural water supply and environmental sanitation project. Dehradun: Govt. Uttaranchal,.

56. Unicef (undated) Immediate Needs: Malawi Foods. Unicef. 6 p.

57. Unicef (2007) Life saving hygiene programme for students in rural Tajikistan. Unicef.

58. Saksena D, Mishra, A., Saade, C., Winger, C. and Ramlow, R. Expanding water purification practices among India's poor: POUZN Project experiences and lessons.: AED Centre for Private Sector Health Initiatives.

59. NIOEH (2007) Setting up of a model for community drinking water quality management system in selected villages. Ha Noi: National Institute of Occupational and Environmental Health.

60. Byleveld P (2000) Assessment of the colilert field test kit system for the detection of coliforms and Escherichia coli in remote aboriginal communities in Australia. Gladesville: New South Wales Health Department.

61. Clarke K, Pearce G, Byleveld P (2002) The NSW Colisure Program – Monitoring Drinking Water in Aboriginal Communities. New South Wales Health Unit.

62. Barnett R, Nean K, Miller M, Standen J, Byleveld P (2004) Drinking water quality in Indigenous communities - The NSW Colisure Program and beyond. 5th National Conference on Indigenous Environmental Health. Terrigal: Dept Health & Aging.

63. Cooke R, Sharp E (2007) Water as a basic human right: Striving for equity in remote, rural and Indigenous South Australian water supplies. Public Health Bulletin South Australia 4: 6-9.

64. IDRC (1996) Presence/Absence Programme: Evaluation Report. Ottawa: International Development Research Centre.

65. Live and Learn (2006) Introductory guidelines for community mobilisation in the Maldives. Volume 3. Male: Live and Learn.

66. van der Tak S (undated) Innovative Actions for Community-based Water Management and Education. Live & Learn.

67. Tun TN (2003) Arsenic contamination of water sources in rural Myanmar. 29th WEDC International Conference: Towards the millenium development goals. Abuja, Nigeria: WEDC.

68. Venkatachalam L (2008) Role of Information on Water Quality in Influencing Household Willingness to Pay: A case study in a peri-urban context. The Icfai University Journal of Urban Policy 3: 7-15.

69. Shrestha RR, Shrestha MP, Upadhyay NP, Pradhan R, Khadka R, et al. (2003) Groundwater arsenic contamination, its health impact and mitigation program in Nepal. Journal of Environmental Science and Health - Part A Toxic/Hazardous Substances and Environmental Engineering 38: 185-200.

70. Mimi ZA, Salman R, Mimi ZA, Salman R (2008) Water quality and improving hygienic practices of the rural community in the vicinity of Ramallah, West Bank, Palestine. International Journal of Environmental Health Research 18: 375-385.

71. Ahmed M, Jakariya M, Quaiyum M, Mahmud SN (2002) An implementation guide for the arsenic mitigation program. Dhaka: BRAC.

72. Hadi A (2001) Promoting health knowledge through micro-credit programmes: experience of BRAC in Bangladesh. Health Promotion International 16: 219-227.

73. Jakariya M (2003) The use of alternative safe water options to mitigate the arsenic problem in Bangladesh: community perspective. Dhaka: BRAC.

74. Schoenfeld A (2005) Area, Village, and Household Response to Arsenic Testing and Labeling of Tubewells in Araihazar, Bangladesh. New York: Columbia University. 32 p.

75. Mishra VK, Singh AK (2008) Institutionalizing community led action for sanitary survey. 33rd WEDC International Conference. Accra, Ghana.

76. Health Canada (2008) First Nations, Inuit and Aboriginal Health: Drinking Water Quality. Health Canada.

77. Indian and Northern Affairs Canada (2003) National Assessment of Water and Wastewater Systems in First Nations Communities. Indian and Northern Affairs Canada.

78. MacMillan N (1993) Indigenous peoples test the water. IRDC Reports 21.

79. Howard G, Luyima P (1999) Report on water's supply surveillance in ten selected urban areas of Uganda. Loughborough: Water Engineering and Development Centre.

80. Howard G (2002) Effective Approaches to Water Supply Surveillance in Urban areas of Developing Countries. Guildford: University of Surrey. 387 p.

81. AED (2005a) Point-of-use water disinfection and zinc treatment project (POUZN). AED.

82. AED (2005b) News: POUZN to reach over one million with point-of-use water disinfection in India. AED.

83. Sanchez A, Dutka B (1998) Community-Based Water Quality Monitoring (WQM) for Panama Rural Health and Water Programs. Ontario: IDRC.

84. Unicef (2003) The Aral Sea and drought. Unicef.

85. Unicef (undated) A guidebook on School Sanitation & Hygiene Promotion Project. Tajikistan: Unicef.
