# Supplementary material for: Dissemination of Drinking Water Contamination Data to Consumers: A Systematic Review of Impact on Consumer Behaviors
Source: PLoS One. 2011 Jun 27;6(6):e21098. doi: 10.1371/journal.pone.0021098 (PMC3124476; doi:10.1371/journal.pone.0021098)
Supplement: Table S1 — Detail of Projects Meeting Intervention Criteria Not Included in Systematic Review. (DOC) [file pone.0021098.s003.doc]

## Table S1 Detail of Projects Meeting Intervention Criteria Not Included in Systematic Review

| **Publications**  **/Reports** | **Population** | **Intervention** | **Reason for Exclusion** |
| --- | --- | --- | --- |
| *Arsenic Contamination Studies* | | | |
| BRAC  [71,72,73] | SW  Bangladesh  Rural areas  25% wells exceed national safe standard for As (50µg/ltr) | Wells tested for arsenic and information returned through well-labelling as safe/unsafe, public education campaign  and face to face meetings; alternative safe wells provided | Although a cohort study, no baseline data were recorded on outcomes of interest, therefore only cross-sectional data at follow up |
| NIOEH [59] | Vietnam  97%, 44% and 3% of water samples in each community exceed Vietnamese standards for As (10ppb) | Local leaders in 3 villages trained to test water quality. Pamphlets distributed to all households and radio broadcast informed people about “quality of water and risk of water contamination” | Although information about water quality was certainly included in the intervention, it is not clear whether this included specific information about results of tests for particular sources. |
| Schoenfeld [74] | Bangladesh  Rural area  Approximately 1/3 of the country has wells which exceed the national standard for As (50µg/ltr) | Well testing and labelling as safe / unsafe | Cross-sectional design |
| Shrestha [69] | Nepal  5% of wells exceeded national standard for As (50µg/ltr)  23% of wells exceeded WHO standard for As (10µg/ltr) | Reports on testing of wells, and describes some dissemination of information taking place (wells labelled with a cross or tick denoting contaminated wells) | No evaluation of information dissemination reported here |
| Tet Nay Tun [67] | Myanmar  35% of wells exceed national standard for As (50µg/ltr) | Reports on implementation of a community-based pilot arsenic action project which included the use of village volunteers to test and communicate results of testing to villages | No relevant outcomes reported |
| *Microbiological Contamination Studies* | | | |
| Govt of India [54,55,75] | India  Rural areas of 3 states: Uttaranchal /Uttarakhand, Orissa & Uttar Pradesh | Community monitoring of drinking water quality using H2S tests. Separate projects initiated by different Indian state Governments in response to new Govt. of India policies. | No systematic evaluations of the impact of interventions could be located |
| Health Canada [64,76,77,78] | Rural First Nations  Communities, Canada | Local personnel were trained and field laboratories set up in communities so that they were equipped to test their water for microbial contamination | No systematic evaluation of the impact of intervention undertaken, only anecdotal reports of outcome |
| Howard [79,80] | Uganda  Urban areas  Across 9 urban areas, the average number of samples exceeding the standard of total coliforms <1cfu/100ml (same for national and WHO standards) ranged from 0% to 66% | Household water tested for thermotolerant coliforms and results returned to householders –quarterly testing over one year | Repeat assessment of outcomes of interest not taken |
| Klink [49] | Uganda  Rural area  90% water samples were contaminated with E.coli (exceeding WHO standard of 0 per 100ml) | The use of water quality testing was used to inform the design of an education package. Village leaders themselves tested samples | The water testing intervention was the outcome of this study, so no evaluation of its use was included |
| Live and Learn pilot [65,66] | Maldives  Rural area  Gastro-related illnesses reported | Local people were trained to use H2S test kits, conduct water monitoring and report  their results to a community management committee | No systematic evaluation of the pilot undertaken, participants asked to report what they had learnt. |
| Malteser International [50,51] | Sri Lanka | H2S tests used by householders to  monitor their own water quality | No systematic evaluation of the impact of intervention undertaken, only anecdotal reports of the experience of the project included |
| Mimi [70] | Palestine  Of the 50 water samples taken (one from each household water tank), 8 tested positive for total coliforms, 4 for fecal coliforms and 5 for Pseudomonas aeruginosa. | Householders informed about microbial contamination of stored water as part of health education and health promotion campaign | Role of test results in the intervention not clear (only briefly mentioned and role in outcome not explored) and no control or comparison group |
| Nair [52] | Australia  Aboriginal communities in remote rural areas | Water testing kit (H2S) and training materials distributed to communities | No systematic evaluations of the impact of intervention could be located |
| New South Wales Colisure Program [60,61,62,63] | South Australia  30% of water samples tested positive for total coliforms 4% for E.coli | Community-based water quality monitoring using  Colilert or Colisure alongside the normal lab-based testing regime | No systematic evaluation of the impact of pilot undertaken, only anecdotal reports of outcome |
| NIOEH [59] | Vietnam  100%, 89% and 47% of water samples in each community exceed Vietnamese & WHO standards for total coliforms (<1 in 100ml) | Local leaders in 3 villages trained to test water quality. Pamphlets distributed to all households and radio broadcast informed people about  “quality of water and risk of water contamination” | Although information about water quality was certainly included in the intervention, it is not clear whether this included specific information about results of tests for particular sources. |
| Point-of-Use Water Disinfection and Zinc Treatment (POUZN) pilot [58,81,82] | Uttar Pradesh, India Rural and urban areas | H2S tests were used to test water, including some conducted by local residents. Test results were presented and discussed at community meetings.  Affordable and/or free water treatment equipment and supplies was also provided | Incomplete reports available, ongoing project without clear evaluation methods or outcomes described. |
| Sanchez and Dutka [83] | Panama  Rural  Positive H2S tests reported | Local community members were trained as water quality testers and information was shared locally | Excluded  Outcomes of interest for systematic review not recorded, no control villages |
| Sun Water: Nepal [23] | Nepal  80% of the 40 water sources tested positive for total coliforms | Drinking water quality was tested and although neither methods of testing nor  of information return are stated explicitly, it is possible to infer that water quality results were shared with at least some residents | Exclude  No systematic evaluation of the impact of intervention undertaken, only anecdotal reports of the experience of the project included |
| Unicef Uzbekistan[84] | Uzbekistan  A third of population estimated to be drinking unsafe water | Community level water quality monitoring | No systematic evaluations of the impact of intervention could be located. |
| Unicef Tajikistan [57,85] | Tajikistan  Rural areas | Community level water quality monitoring using school as agency | No systematic evaluations of the impact of intervention could be located. |
| Unicef Malawi [56] | Malawi  Rural areas affected by seasonal flood | Water testing kits (H2S) distributed to households for testing their drinking water during flood emergency | No systematic evaluations of the impact of intervention could be located. |
| Venkatachalam, 2008 [68] | Tamil Nadu, India  Urban settings | Study of how providing relevant, ‘additional’ information on water quality influences households’ willingness to pay | Outcomes of interest for systematic review not recorded, no control data  Full text not available |
| WHO Darfur [53] | Darfur, Sudan  Refugee camp | Water testing kits (H2S) distributed to agencies managing refugee camps for testing drinking water | No systematic evaluations of the impact of intervention could be located. |
